# Anthocidins A–D, New 5-Hydroxyanthranilic Acid Related Metabolites from the Sea Urchin-Associated Actinobacterium, *Streptomyces* sp. HDa1

**DOI:** 10.3390/molecules23051032

**Published:** 2018-04-27

**Authors:** Zhi-Kai Guo, Rong Wang, Shi-Quan Chen, Fu-Xiao Chen, Tian-Mi Liu, Ming-Qiu Yang

**Affiliations:** 1 Key Laboratory of Biology and Genetic Resources of Tropical Crops, Ministry of Agriculture, Institute of Tropical Bioscience and Biotechnology, Chinese Academy of Tropical Agricultural Sciences, Haikou 571101, China; 2 Hainan Academy of Ocean and Fisheries Sciences, Haikou 570203, China; helen1982--@163.com (R.W.); breezysmile.c.s.q@163.com (S.-Q.C.); cfx69@163.com (F.-X.C.); ymq5338@163.com (M.-Q.Y.); 3 Hainan Testing Center for the Quality and Safety of Aquatic Products, Haikou 570206, China; ltm600@163.com

**Keywords:** natural products, marine actinomycete, *Streptomyces* sp., anthocidin

## Abstract

Four new 5-hydroxyanthranilic acid related compounds, named anthocidins A–D (**1**–**4**), two known analogues *n*-lauryl 5-hydroxyanthranilate (**5**) and isolauryl 5-hydroxyanthranilate (**6**), together with benzamide (**7**), 3-hydroxy-4-methoxycinnamamide (**8**), and (3*S*-*cis*)-hexahydro-3-[(3,4-dihydroxyphenyl)methyl]pyrrolo[1,2-*a*]pyrazine-1,4-dione (**9**), were isolated from the fermentation broth of the marine-derived actinomycete, *Streptomyces* sp. HDa1, which was isolated from the gut of a sea urchin, *Anthocidaris crassispina*, collected from Hainan Island, China. The structures of these secondary metabolites were elucidated on the basis of their 1D and 2D-NMR and mass spectroscopic data, and anthocidin A was confirmed by single-crystal X-ray diffraction with Cu Kα radiation. Anthocidins A–D (**1**–**4**) feature an acetyl group substitution at the amino group and varying alkyl side chains at the carboxyl group of 5-hydroxyanthranilic acid, and compound **5** was isolated as a natural product for the first time. The cytotoxic and antibacterial activity of compounds **1**–**9** were evaluated.

## 1. Introduction

**Figure 1:**
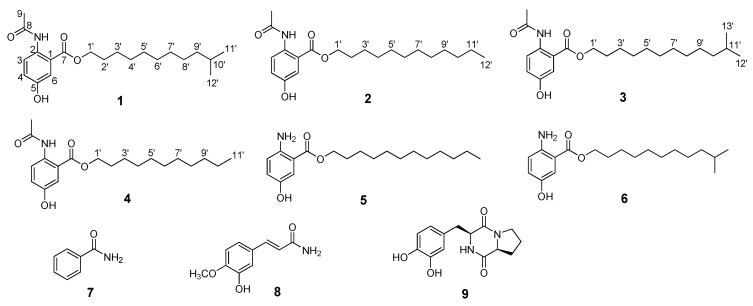
The structures of compounds **1**–**9**.

Natural products from actinomycetes have played a key role in drug discovery for the treatment of human diseases as exemplified by the immunosuppressant rapamycin; the antifungal agent nystatin; and the antibiotics tetracyclines, erythromycin, and vancomycin [1]. Despite the previous success of pharmaceutical compounds from actinomycetes, the constant need for the discovery of bioactive natural products has prompted the microbial natural product chemists to apply diverse strategies to identify novel secondary metabolites. One strategy is the exploration of the actinomycetes that inhabit un- or under-explored environments such as marine ecosystems. Marine-sourced actinomycetes have been proven to be rich sources of structurally diverse and biological active natural products [2,3]. A number of bioactive secondary metabolites featuring interesting structural properties have been discovered recently in marine-derived actinomycetes, such as tetrocarcin Q [4], fluostatins M–Q [5], succinilenes A–D [6], strepchazolins A and B [7], and a new napyradiomycin analogue [8], showing various potential antibacterial, antifungal, and antitumor activities. The under-explored marine animal-symbiont associations could provide a tremendous opportunity for the natural products discovery [9,10,11]. In our continuing efforts to search for novel bioactive natural products from marine microbes [12,13,14], recently we isolated an actinomycete strain *Streptomyces* sp. HDa1 from the gut of a sea urchin, *Anthocidaris crassispina*, collected from Hainan Island, China. Subsequent chemical study on the large-scale fermentation broth of this strain led to the discovery of four new 5-hydroxyanthranilic acid related compounds, anthocidins A–D (**1**–**4**), two known analogues *n*-lauryl 5-hydroxyanthranilate (**5**) and isolauryl 5-hydroxyanthranilate (**6**) [15], and benzamide (**7**) [16], 3-hydroxy-4-methoxycinnamamide (**8**) [17], and (3*S*-*cis*)-hexahydro-3-[(3,4-dihydroxyphenyl)methyl]pyrrolo[1,2-*a*]pyrazine-1,4-dione (**9**) [18] (Figure 1). Unlike the known analogues **5** and **6**, anthocidins A–D feature varying alkyl side chains and possess an *N*-acetyl group at the C-2 position. In this paper, we describe the isolation and structure elucidation of these four new 5-hydroxyanthranilic acid derivatives as well as their cytotoxic and antibacterial activity.

## 2. Results

**Figure 2:**
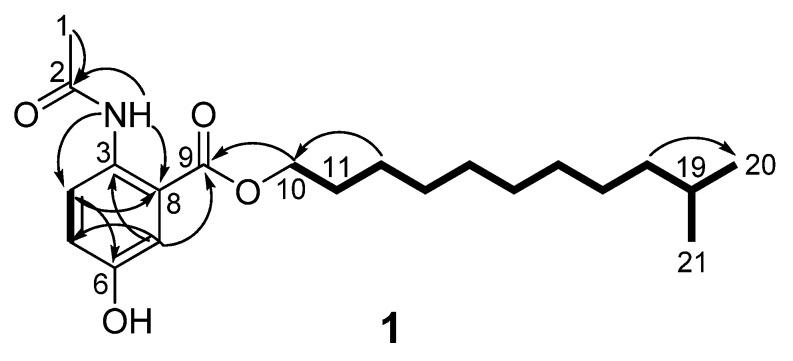
Key ^1^H–^1^H COSY (bold lines) and HMBC (arrows) correlations of **1**.

**Figure 3:**
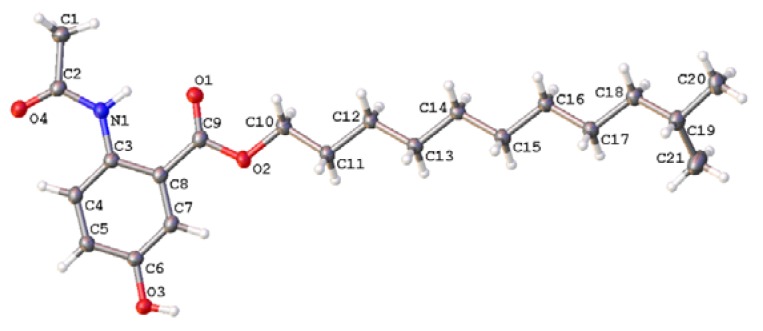
X-ray single-crystal structure of **1**.

Anthocidin A (**1**) was obtained as light yellow needles. Its molecular formula was determined as C_21_H_33_NO_4_ on the basis of the high resolution electrospray ionization mass spectroscopy (HRESIMS) data at *m/z* 386.2309 [M + Na]^+^ (calcd for C_21_H_33_NO_4_Na, 386.2308) together with its NMR data (Table 1). In the ^1^H-NMR spectrum, the splitting patterns for three coupled aromatic protons (*δ*_H_ 8.53, d, *J* = 9.1 Hz, H-3; 7.15, dd, *J* = 9.1, 3.0 Hz, H-4; 7.55, d, *J* = 3.0 Hz, H-6) indicated the presence of a 1,2,4-trisubstituted benzene ring. The ^1^H-NMR data of **1** also showed three methyl groups (*δ*_H_ 2.25, 3H, s, H_3_-9; 0.87, 6H, d, *J* = 6.6 Hz, H_3_-11′ and H_3_-12′) and one exchangeable proton (*δ*_H_ 10.93, s, 2-NH). The ^13^C and DEPT135-NMR spectra revealed the presence of two carbonyl carbons (*δ*_C_ 169.4 and 168.1), six aromatic carbons (*δ*_C_ 116.8–151.7) (including three methine and three quaternary carbons), one sp^3^ methine (*δ*_C_ 27.9), nine sp^3^ methylene (*δ*_C_ 26.0–65.7) and three methyl carbons (*δ*_C_ 22.7–25.2). The HSQC spectrum of **1** allowed all protons to be assigned to their respective carbons and the structure of anthocidin A (**1**) was elucidated by the interpretation of its HMBC and ^1^H-^1^H COSY correlations (Figure 2). In the HMBC spectrum, the correlations from the aromatic signal H-3 to C-1 (*δ*_C_ 116.8) and C-5 (*δ*_C_ 151.7), from the aromatic signal H-6 to C-2 (*δ*_C_ 134.1), C-4 (*δ*_C_ 121.8) and the carboxyl carbon C-7 (*δ*_C_ 168.1) and from 2-NH to C-1 and C-3 (*δ*_C_ 122.1) indicated the presence of a 1,2,4-trisubstituted benzene, which could be also supported by the ^1^H-^1^H COSY correlation from H-3 to H-4. In addition, the HMBC correlations from 2-NH and H_3_-9 to the amide carbon C-8 (*δ*_C_ 169.4) suggested an *N*-acetyl group at the C-2 position. The last partial structure was identified as an isolauryl alcohol moiety which was deduced by the interpretation of the clear ^1^H-^1^H COSY correlations from two terminal methyl groups (H-11′ and H-12′) to a methine proton H-10′ (*δ*_H_ 1.53, m), from H-9′ (*δ*_H_ 1.16, m) to H-10′ and H-8′ (*δ*_H_ 1.20–1.38, m), from H-2′ (*δ*_H_ 1.77, m) to H-1′ (*δ*_H_ 4.31, t, *J* = 6.7 Hz) and H-3′ (*δ*_H_ 1.45, m), and the overlapped COSY correlations among the methylene protons. The connection of the isolauryl alcohol moiety to the C-7 on the 1,2,4-trisubstituted benzene ring through an oxygen bridge was secured by the HMBC correlations from the oxygenated methylene protons H-1′ to C-7. In addition, analysis of the HRESIMS and NMR data revealed one hydroxyl group could be located at C-5 on the 1,2,4-trisubstituted benzene ring. Finally, the structure of anthocidin A (**1**) was elucidated as shown, which was also confirmed by single-crystal X-ray crystallographic analysis in a Cu Kα radiation in low temperature (Figure 3).

Anthocidin B (**2**) was obtained as light yellow needles with the molecular formula of C_21_H_33_NO_4_ as deduced from the positive HRESIMS data at *m/z* 386.2310 [M + Na]^+^ (calcd for C_21_H_33_NO_4_Na, 386.2308)), ^1^H and ^13^C-NMR data. The ^1^H and ^13^C-NMR data of **2** was almost identical to those of **1** (Table 1). Extensive comparative analysis of the MS and ^1^H, ^13^C, DEPT135, and HSQC NMR data of **2** with those of **1** disclosed the presence of one additional methylene group and the absence of one terminal methyl group in the lipophilic chain of **2**. Therefore, the structure of **2** was determined as an analogue of **1** with a lauryl alcohol chain by complete analysis of the HSQC, 1H-1H COSY, and HMBC spectra. 

Anthocidin C (**3**) was isolated as a light yellow solid and had the molecular formula C_22_H_35_NO_4_ by analysis of HRESIMS data at *m/z* 400.2466 [M + Na]^+^ (calcd for C_22_H_35_NO_4_Na, 400.2464) and NMR data (Table 1). Its mass data was found to be 14 Da higher than that of **1**. The ^1^H and ^13^C-NMR data of **3** was almost identical to those of **1** except for one more methylene group in the lipid chain of **3**. Thus, the structure of **3** was deduced to be an analogue of **1** with an isotridecyl alcohol chain by extensive NMR analysis.

Anthocidin E (**4**), isolated as a light yellow amorphous powder, gave a molecular formula of C_20_H_31_NO_4_ based on the HRESIMS data at *m/z* 372.2154 [M + Na]^+^ (calcd for C_20_H_31_NO_4_Na, 372.2151), and ^1^H and ^13^C-NMR data (Table 2). Comparison of the NMR data of **4** with those of **2** revealed that they are almost identical except for absence of one methylene group in **4**. On the basis of the 2D NMR data including the HSQC, ^1^H–^1^H COSY, and HMBC data of **4**, its structure was unambiguously assigned as shown, possessing an undecyl alcohol side chain.

Compounds **5** and **6** were characterized as *n*-lauryl 5-hydroxyanthranilate, a chemically-synthesized 5-hydroxyanthranilate ester, and isolauryl 5-hydroxyanthranilate, respectively, by comparison of their NMR data with those reported data [15]. The literature also reported that these compounds possessed potent in vitro 5-lipoxygenase inhibitory activity. Herein, compound **5** was isolated and reported as a natural product for the first time. 

Compounds **1**–**9** were screened for their in vitro antibacterial activities against a variety of bacteria including *Staphylococcus aureus*, *Streptococcus pyogenes*, *Bacillus subtilis*, *Vibrio harveyi*, and *Vibrio alginolyticus* at a concentration of 10 mg/mL. As a result, only compound **8** showed weak activity against the Gram-positive bacterium *Bacillus subtilis* with inhibition zone of 3 mm, while compound **9** displayed weak activity against the Gram-negative bacterium *Vibrio harveyi* with inhibition zone of 1.5 mm. Due to their weak activity at a high concentration, we did not determine the minimum inhibitory concentration. Also, their in vitro cytotoxic activities against the human melanoma cell line A375 and human ovarian carcinoma cell line CaoV3 were tested. However, none of these natural products exhibited potent cytotoxicity against these human cancer cell lines at a concentration of 10 µM. 

## 3. Materials and Methods 

### 3.1. General Experimental Procedures

NMR data were recorded in CDCl_3_ using a Bruker DRX-600 spectrometer (600 MHz for ^1^H-NMR and 150 MHz for ^13^C-NMR) with TMS (tetramethylsilane) as the internal standard (*δ* in ppm, *J* in Hz) (Bruker Corporation, Karlsruhe, Germany). High resolution electrospray ionization mass spectra were obtained on an Agilent 6210 TOF LC-MS spectrometer (Agilent Technologies Inc., Palo Alto, CA, USA). Silica gel (200–300 mesh, Qingdao Marine Chemical Factory, Qingdao, China) and Sephadex LH-20 (GE Healthcare Bio-Sciences AB, Uppsala, Sweden) were used for column chromatography. Semipreparative reverse-phase (RP) HPLC was conducted on an Hitachi HPLC system (Hitachi High-Technologies Corporation, Tokyo, Japan) consisting of a Hitachi L-7110 pump (Hitachi) and a Hitachi L-7420 UV–vis detector equipped with a Hypersil RP-C18 column (5 µm, 250 × 10.0 mm, Thermo Fisher Scientific, Waltham, MA, USA).

### 3.2. Strain Isolation and Cultivation

The strain HDa1 was isolated by one of the authors (R.W.) from the gut of a sea urchin, *Anthocidaris crassispina*, collected from Hainan Island, China, using the ISP4 agar medium (consisting of 10.0 g/L soluble starch, 1.0 g/L K_2_PO_4_, 1.0 g/L MgSO_4_·7H_2_O, 1.0 g/L NaCl, 2.0 g/L (NH_4_)_2_SO_4_, 2.0 g/L CaCO_3_, 0.001 g/L FeSO_4_·7H_2_O, 0.001 g/L MnCl_2_·7H_2_O, 20 g/L agar, and deionized water, pH 7.2) supplemented with a final concentration of 50 µg/mL potassium dichromate. It has been identified as *Streptomyces* sp. by 16S rRNA sequence analysis (GenBank accession number is MG745333). A voucher specimen (HNHDa1) has been deposited in the Institute of Tropical Bioscience and Biotechnology, Chinese Academy of Tropical Agricultural Sciences. The strain was cultivated on ISP4 agar plates at 28 °C. After five days, the spores were inoculated into 10 500 mL-Erlenmeyer flasks, each containing 100 mL of sterile seed medium (Tryptone soy broth, 30 g/L) and cultivated for 3 days at 28 °C with 160 rpm/min. Then, 10 mL of the seed cultures were inoculated into 100 × 1000 mL-Erlenmeyer flasks with 200 mL of a fermentation medium consisting of 4 g/L yeast extract, 10 g/L malt extract, and 4 g/L glucose (pH 7.2) and fermented on a rotary shaker with 140 rpm/min at 28 °C for 10 days. 

### 3.3. Extraction and Purification

The entire filtrate of the fermentation broth (about 20 L) was harvested and 4% XAD-16N resin was added. Then the mixture was oscillated on a rotary shaker with 160 rpm/min for 4 h. After that, the resin was collected and extracted four times with methanol at room temperature. Subsequently, the methanol extract was evaporated to dryness under reduced pressure to yield a crude extract (8.0 g), which was then fractionated by silica gel column chromatography (CC) using a gradient elution of petroleum ether/EtOAc (*v*/*v*, 100:0, 50:1, 20:1, 10:1, 5:1, 2:1, 1:1, and 0:100) to give eight fractions (Fr.1–Fr.8). Fr.5 (petroleum ether/EtOAc, *v*/*v*, 5:1) was subsequently subjected to ODS CC with a gradient of MeOH/H_2_O (*v*/*v*, 30:70, 40:60, 50:50, 60:40, 70:30, and 100:0) to give six subfractions (Fr.5.1–Fr.5.6). Fr.5.3 (MeOH/H_2_O, 50:50) was further purified by semipreparative RP-HPLC to yield compounds **1** (6.5 mg), **2** (5.1 mg), **3** (3.0 mg), and **7** (2.3 mg). Fr.6 (petroleum ether/EtOAc, *v*/*v*, 2:1) was subjected to Sephadex LH-20 CC using MeOH as eluents to give eight subfractions, which were further purified by semipreparative RP-HPLC to yield compounds **4** (2.0 mg) and **8** (4.1 mg). Fr.7 (petroleum ether/EtOAc, *v*/*v*, 1:1) was subjected to ODS CC with a gradient of MeOH/H_2_O (*v*/*v*, 30:70, 40:60, 50:50, 60:40, 70:30, and 100:0) to give six subfractions. Fr.5.4 (MeOH/H_2_O, 60:40) was further purified by Sephadex LH-20 CC eluted by MeOH and finally by semipreparative RP-HPLC to generate compounds **5** (6.0 mg), **6** (8.2 mg), and **9** (3.2 mg).

### 3.4. X-ray Single-Crystal Data of **1**

The crystals of **1** were obtained by crystallization from a solution of MeOH/CH_2_Cl_2_ (*v*/*v*, 1:1). The single crystal X-ray diffraction data was obtained on a Bruker APEX-II diffractometer with Cu K*α* radiation (*λ* = 1.54178 Å) at 130 K. The structure was solved using the program SHELXS-97 and refined by full-matrix least-squares on *F^2^*. Crystal data of compound **1** have been deposited with the Cambridge Crystallographic Data Center (deposition no. CCDC 1814418), which can be obtained free of charge via www.ccdc.cam.ac.uk/data_request/cif.

Crystal data for **1**: molecular formula C_21_H_33_NO_4_, *Mr* = 363.48, monoclinic crystals, *a* = 5.3049 (7) Å, *b* = 15.916 (2) Å, *c* = 24.348 (3) Å, α = 90.00°, β = 97.794(11)°, γ = 90.00°, Z = 4, µ = 0.642 mm^−1^, *F* (000) = 792, and *T* = 130 K; Crystal dimensions: 0.12 × 0.08 × 0.06 mm^3^, Volume = 2055.6 (5) Å^3^, 9576 reflections measured, 3635 independent reflections (*R_int_* = 0.0522), the final *R* indices [*I* > 2*σ*(*I*)] *R_1_* = 0.0519, *wR_2_* = 0.1348, *R* indices (all data) *R_1_* = 0.0701, *wR_2_* = 0.1482. The goodness of fit on *F^2^* was 1.041.

### 3.5. Biological Assays

Cytotoxic activities of compounds **1**–**9** against the human melanoma cell line A375 and human ovarian carcinoma cell line CaoV3 were evaluated with the MTT assay [19]. The antibacterial activity of these compounds were also tested against *Staphylococcus aureus*, *Streptococcus pyogenes*, *Bacillus subtilis*, *Vibrio harveyi*, and *Vibrio alginolyticus*, in accordance with the previously reported method [20]. In the assays, the antibacterial activities were tested using the agar diffusion method with 7 mm paper discs containing 100 µg of compounds and rifampicin as the positive control. All tested compounds were dissolved in dimethyl sulfoxide (DMSO).

## 4. Conclusions

Four new 5-hydroxyanthranilic acid derivatives, anthocidins A–D (**1**–**4**), and *n*-lauryl 5-hydroxyanthranilate (**5**), isolauryl 5-hydroxyanthranilate (**6**), benzamide (**7**), 3-hydroxy-4-methoxycinnamamide (**8**), and (3*S*-*cis*)-hexahydro-3-[(3,4-dihydroxyphenyl)methyl]- pyrrolo[1,2-*a*]pyrazine-1,4-dione (**9**), were isolated from a sea urchin (*Anthocidaris crassispina*)-associated actinomycete, *Streptomyces* sp. HDa1. Their structures were determined by 1D and 2D-NMR and mass spectroscopic data, and anthocidin A was confirmed by single-crystal X-ray diffraction with Cu Kα radiation. Anthocidins A–D feature an acetamide group substitution at the amino group and varying ester chains at the carboxyl group of 5-hydroxyanthranilic acid, and compound **5** was isolated as a natural product for the first time. In the biological assays, compound **8** showed weak activity against the Gram-positive bacterium *Bacillus subtilis*, while compound **9** displayed weak activity against the Gram-negative bacterium *Vibrio harveyi*. However, none of these natural products exhibited potent cytotoxicity against these human cancer cell lines at a concentration of 10 µM. The result showed that the marine animal-symbiont actinomycetes could provide a good opportunity for discovering diverse new or bioactive natural products. 

## Figures and Tables

**Table 1:** ^1^H and ^13^C-NMR data for anthocidins A–C (**1**–**3**) in CDCl_3_. ^a.^

Position	Anthocidin A (1)		Anthocidin B (2)		Anthocidin C (3)		
	*δ*_C_	*δ*_H_ (*J* in Hz)	*δ*_C_	*δ*_H_ (*J* in Hz)	*δ*_C_	*δ*_H_ (*J* in Hz)	
1	116.8, C		116.8, C		116.7, C		
2	134.1, C		134.3, C		135.1, C		
2-NH		10.93, s		10.88, s			10.84, s
3	122.1, CH	8.53, d (9.1)	122.1, CH	8.50, d (9.0)	122.2, CH		8.53, d (8.9)
4	121.8, CH	7.15, dd (9.1, 3.0)	121.8, CH	7.10, dd (9.0, 2.4)	121.9, CH		7.07, dd (8.9, 3.0)
5	151.7, C		151.7, C		150.9, C		
6	116.9, CH	7.55, d (3.0)	116.9, CH	7.52, d (2.4)	116.8, CH	7.50, d (3.0)	
7	168.1, C		168.1, C		168.1, C		
8	169.4, C		169.3, C		169.1, C		
9	25.2, CH_3_	2.25, s	25.3, CH_3_	2.22, s	26.2, CH_3_	2.21, s	
1′	65.7, CH_2_	4.31, t (6.7)	65.7, CH_2_	4.28, t (6.6)	65.8, CH_2_	4.30, t (6.7)	
2′	28.6, CH_2_	1.77, m	28.6, CH_2_	1.75, m	28.7, CH_2_	1.76, m	
3′	26.0, CH_2_	1.45, m	26.0, CH_2_	1.42, m	26.2, CH_2_	1.43, m	
4′	27.4, CH_2_	1.20–1.38, m	29.3 ^c^, CH_2_	1.24–1.36, m	22.0–39.0 ^d^, CH_2_	1.20–1.40, m	
5′	29.3 ^b^, CH_2_	1.20–1.38, m	29.4 ^c^, CH_2_	1.24–1.36, m	22.0–39.0 ^d^, CH_2_	1.20–1.40, m	
6′	29.5 ^b^, CH_2_	1.20–1.38, m	29.5 ^c^, CH_2_	1.24–1.36, m	22.0–39.0 ^d^, CH_2_	1.20–1.40, m	
7′	29.7 ^b^, CH_2_	1.20–1.38, m	29.6 ^c^, CH_2_	1.24–1.36, m	22.0–39.0 ^d^, CH_2_	1.20–1.40, m	
8′	29.9 ^b^, CH_2_	1.20–1.38, m	29.6 ^c^, CH_2_	1.24–1.36, m	22.0–39.0 ^d^, CH_2_	1.20–1.40, m	
9′	39.1, CH_2_	1.16, m	29.7 ^c^, CH_2_	1.24–1.36, m	22.0–39.0 ^d^, CH_2_	1.20–1.40, m	
10′	27.9, CH	1.53, m	39.1, CH_2_	1.24–1.36, m	22.0–39.0 ^d^, CH_2_	1.20–1.40, m	
11′	22.7, CH_3_	0.87, d (6.6)	22.8, CH_2_	1.24–1.36, m	28.1, CH	1.51, m	
12′	22.7, CH_3_	0.87, d (6.6)	14.1, CH_3_	0.87, t (6.8)	19.4, CH_3_	0.85, d (6.4)	
13′					11.5, CH_3_	0.83, d (6.4)	

^a 1^H and ^13^C-NMR data were obtained at 600 and 150 MHz, respectively. *δ* in ppm. ^b–d^ interchangeable.

**Table 2:** ^1^H and ^13^C-NMR data for anthocidin D (**4**) in CDCl_3_. ^a^

Position	Anthocidin D (4)				
	*δ*_C_	*δ*_H_ (*J* in Hz)	Position	*δ*_C_	*δ*_H_ (*J* in Hz)
1	116.4, C		1′	65.7, CH_2_	4.30, t (6.7)
2	135.2, C		2′	28.6, CH_2_	1.77, m
2-NH		10.81, s	3′	26.0, CH_2_	1.43, m
3	122.1, CH	8.56, d (9.0)	4′	29.5 ^b^, CH_2_	1.20–1.40, m
4	121.7, CH	7.04, dd (9.0, 3.0)	5′	29.6 ^b^, CH_2_	1.20–1.40, m
5	150.4, C		6′	29.6 ^b^, CH_2_	1.20–1.40, m
5-OH			7′	29.3 ^b^, CH_2_	1.20–1.40, m
6	116.5, CH	7.48, d (3.0)	8′	29.2 ^b^, CH_2_	1.20–1.40, m
7	167.9, C		9′	31.9, CH_2_	1.20–1.40, m
8	168.8, C		10′	22.7, CH_2_	1.20–1.40, m
9	25.3, CH_3_	2.21, s	11′	14.1, CH_3_	0.88, t (7.0)

^a 1^H and ^13^C-NMR data were obtained at 600 and 150 MHz, respectively. *δ* in ppm. ^b^ interchangeable.

## Supplementary Materials

The 1D and 2D-NMR spectra for compounds **1**–**4** are available online.
