# Supplementary material for: Anthocidins A–D, New 5-Hydroxyanthranilic Acid Related Metabolites from the Sea Urchin-Associated Actinobacterium, Streptomyces sp. HDa1
Source: Molecules. 2018 Apr 27;23(5):1032. doi: 10.3390/molecules23051032 (PMC6102551; doi:10.3390/molecules23051032)

Supporting Information

Anthocidins A-D, New 5-Hydroxyanthranilic Acid Related Metabolites from the Sea Urchin-Associated Actinobacterium, *Streptomyces* sp. HDa1

Figure S1. ^{1}H-NMR (600 MHz, $CDCl_3$) spectrum of compound **1**

Figure S2. ^{13}C-NMR (150 MHz, $CDCl_3$) spectrum of compound **1**

Figure S3. DEPT135 spectrum of compound **1** in $CDCl_3$

Figure S4. HSQC spectrum of compound **1** in $CDCl_3$

Figure S5. ^{1}H-^{1}H COSY spectrum of compound **1** in $CDCl_3$

Figure S6. HMBC spectrum of compound **1** in $CDCl_3$

Figure S7. ^{1}H-NMR (600 MHz, $CDCl_3$) spectrum of compound **2**

Figure S8. ^{13}C-NMR (150 MHz, $CDCl_3$) spectrum of compound **2**

Figure S9. DEPT135 spectrum of compound **2** in $CDCl_3$

Figure S10. HSQC spectrum of compound **2** in $CDCl_3$

Figure S11. ^{1}H-^{1}H COSY spectrum of compound **2** in $CDCl_3$

Figure S12. HMBC spectrum of compound **2** in $CDCl_3$

Figure S13. ^{1}H-NMR (600 MHz, $CDCl_3$) spectrum of compound **3**

Figure S14. ^{13}C-NMR (150 MHz, $CDCl_3$) spectrum of compound **3**

Figure S15. HSQC spectrum of compound **3** in $CDCl_3$

Figure S16. ^{1}H-NMR (600 MHz, $CDCl_3$) spectrum of compound **4**

Figure S17. ^{13}C-NMR (150 MHz, $CDCl_3$) spectrum of compound **4**

Figure S18. DEPT135 spectrum of compound **4** in $CDCl_3$

Figure S19. HSQC spectrum of compound **4** in $CDCl_3$

Figure S20. ^{1}H-^{1}H COSY spectrum of compound **4** in $CDCl_3$

Figure S21. HMBC spectrum of compound **4** in $CDCl_3$

Figure S1. ^{1}H-NMR (600 MHz, $CDCl_3$) spectrum of compound **1**

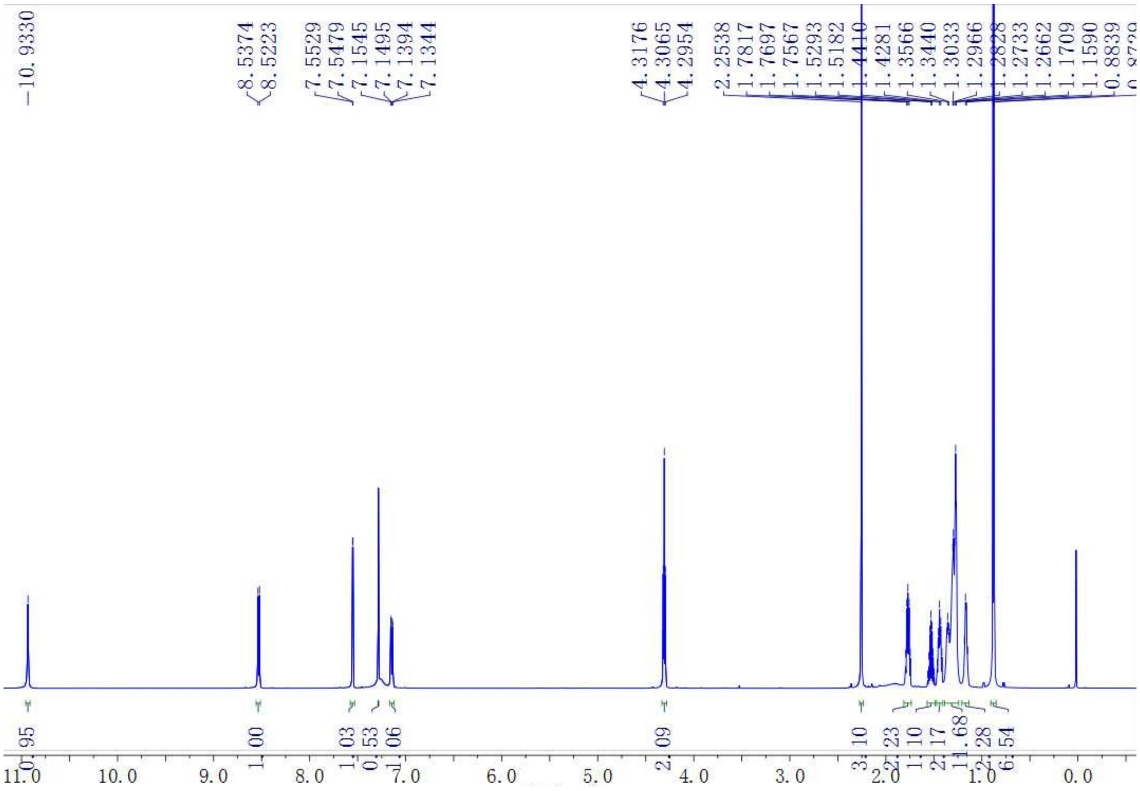

Figure S2. ^{13}C-NMR (150 MHz, $CDCl_3$) spectrum of compound **1**

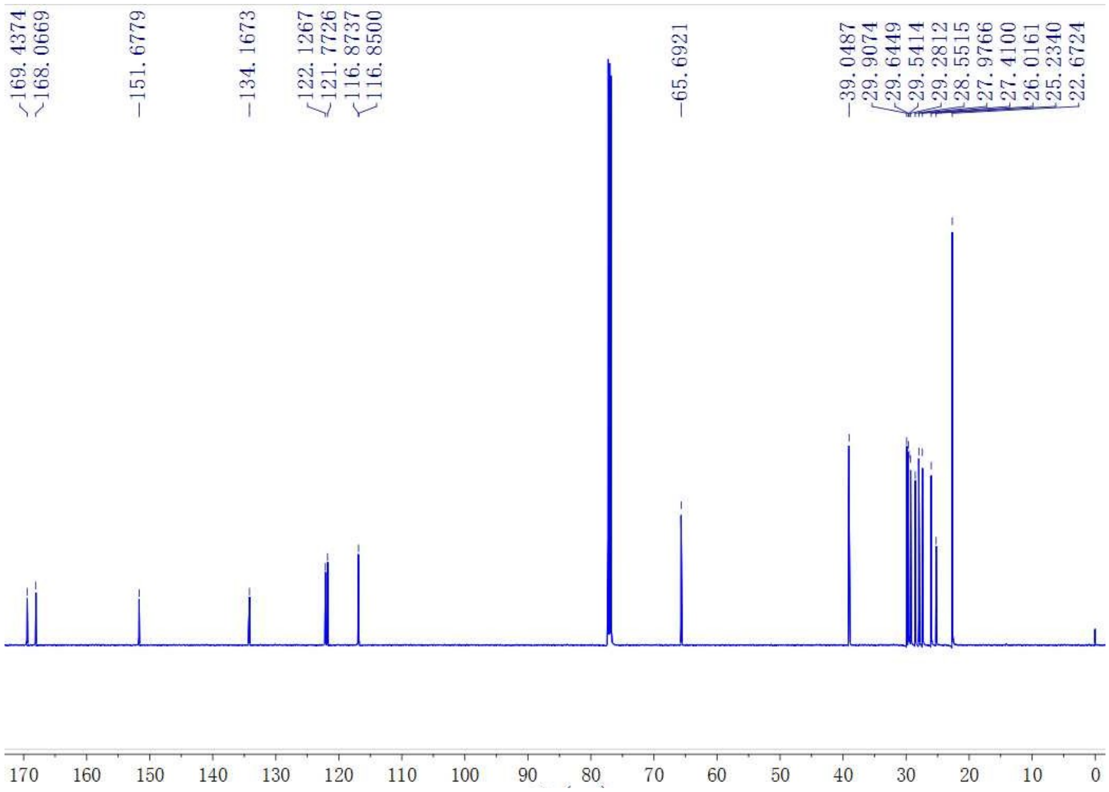

Figure S3. DEPT135 spectrum of compound **1** in $CDCl_3$

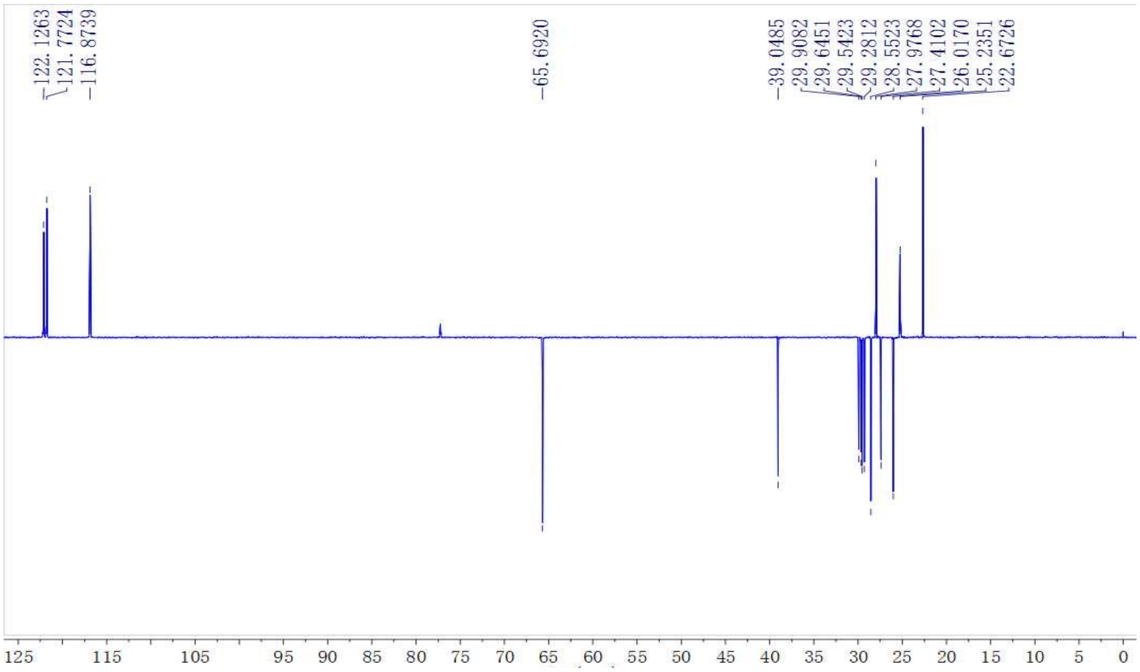

Figure S4. HSQC spectrum of compound **1** in $CDCl_3$

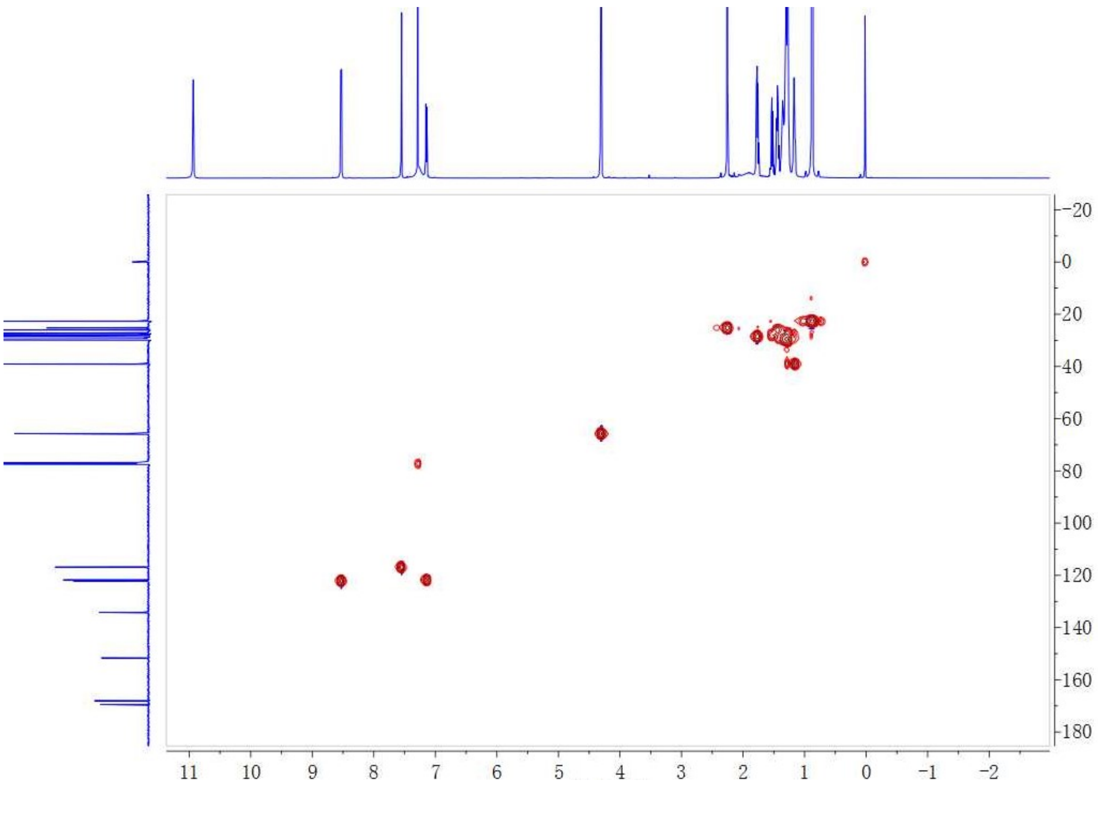

Figure S5. ^1H-^1H COSY spectrum of compound **1** in $CDCl_3$

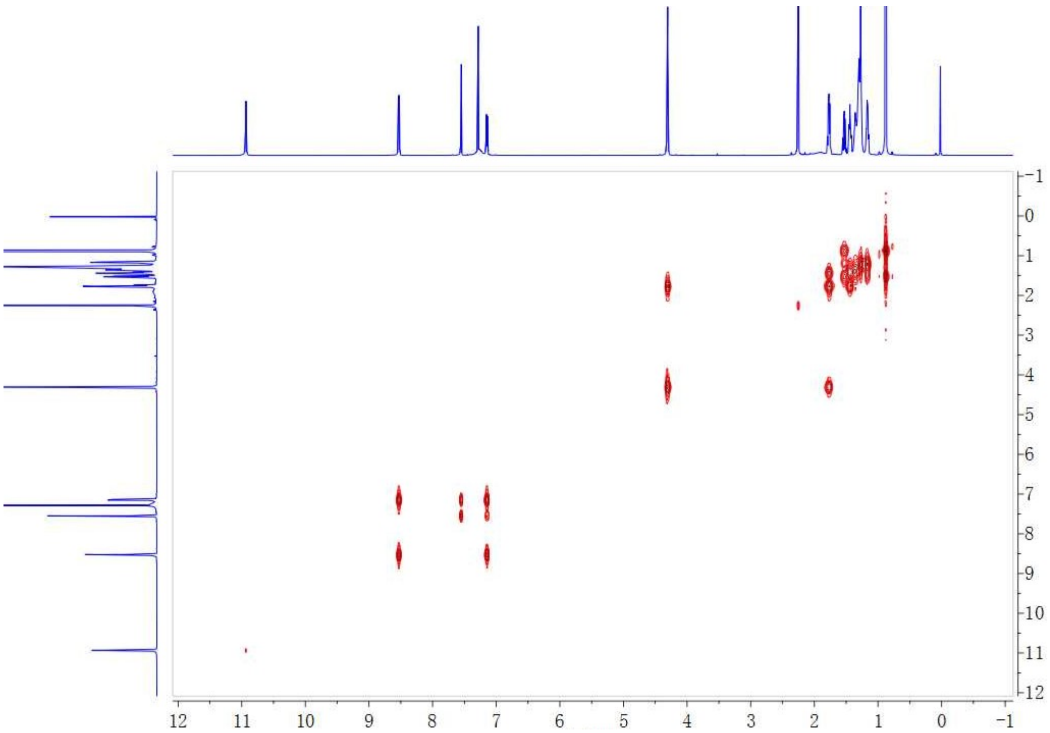

Figure S6. HMBC spectrum of compound **1** in $CDCl_3$

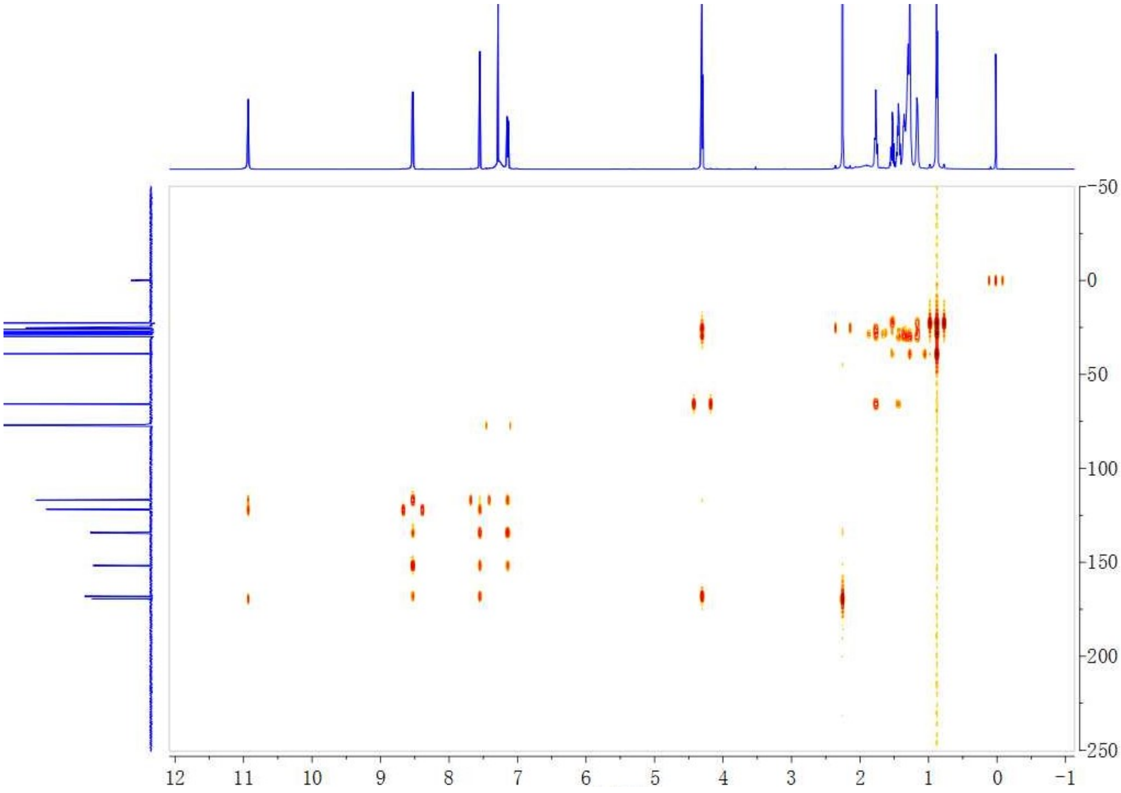

Figure S7. ^{1}H-NMR (600 MHz, $CDCl_3$) spectrum of compound **2**

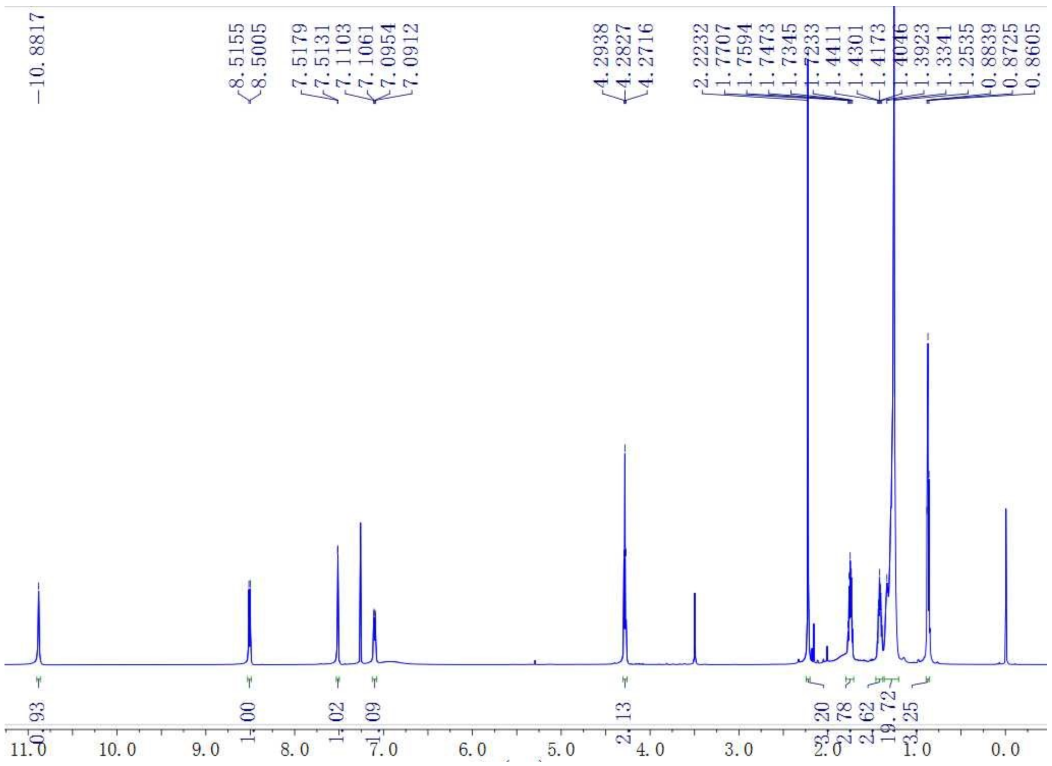

Figure S8. ^{13}C-NMR (150 MHz, $CDCl_3$) spectrum of compound **2**

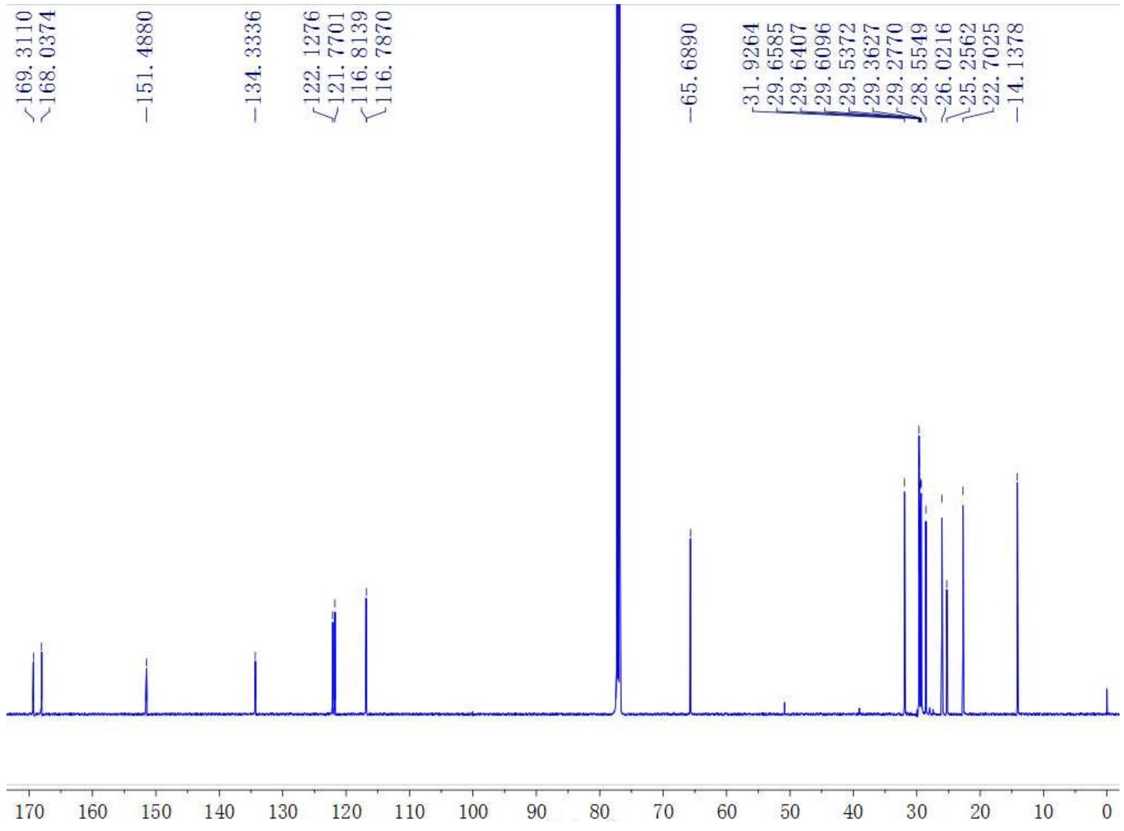

Figure S9. DEPT135 spectrum of compound **2** in $CDCl_3$

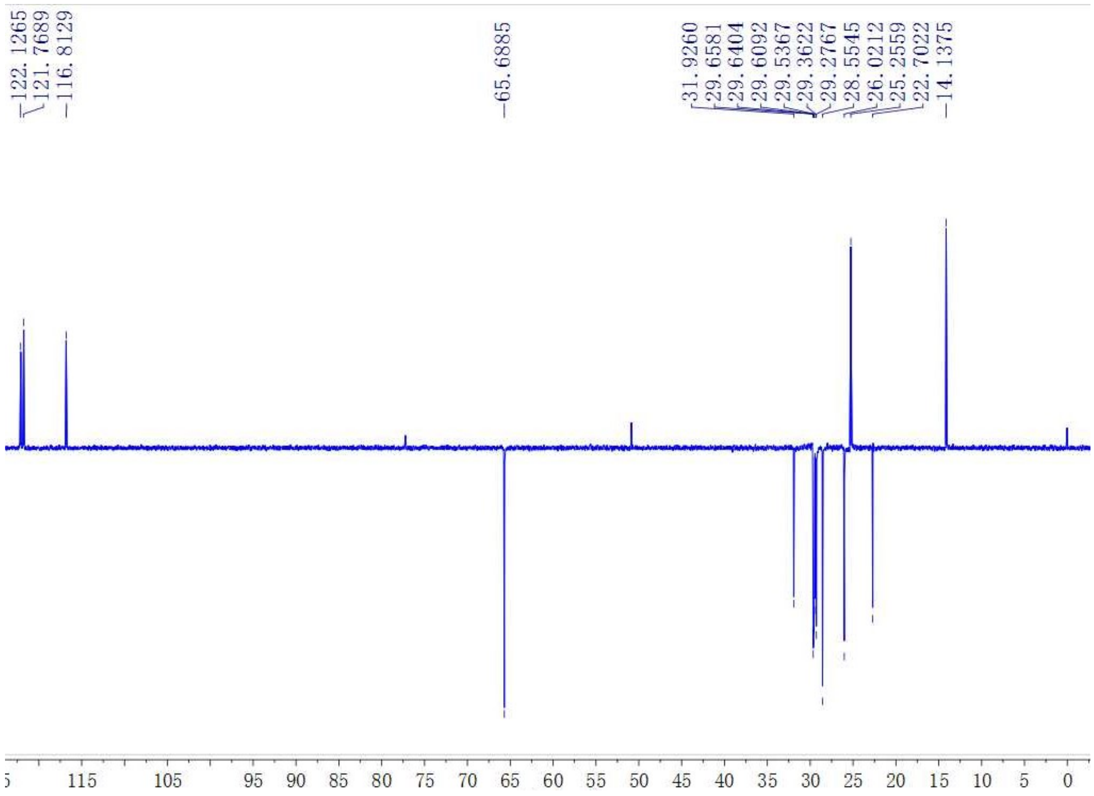

Figure S10. HSQC spectrum of compound **2** in $CDCl_3$

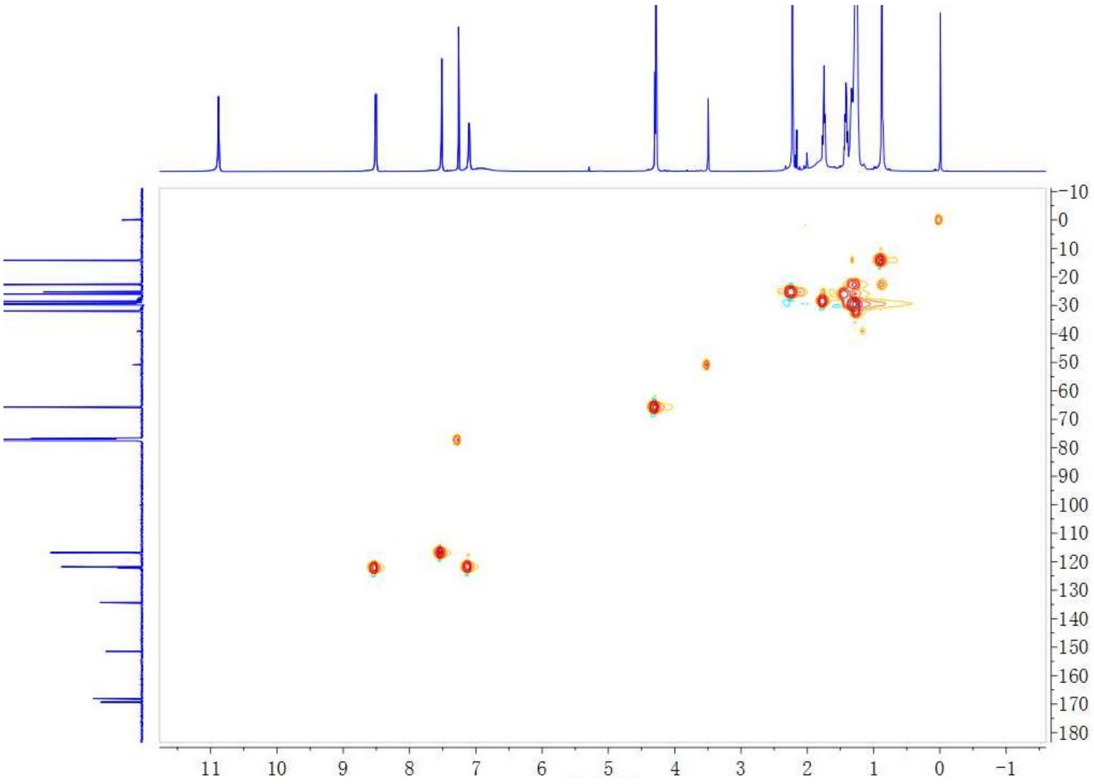

Figure S11. ^{1}H-^{1}H COSY spectrum of compound **2** in $CDCl_3$

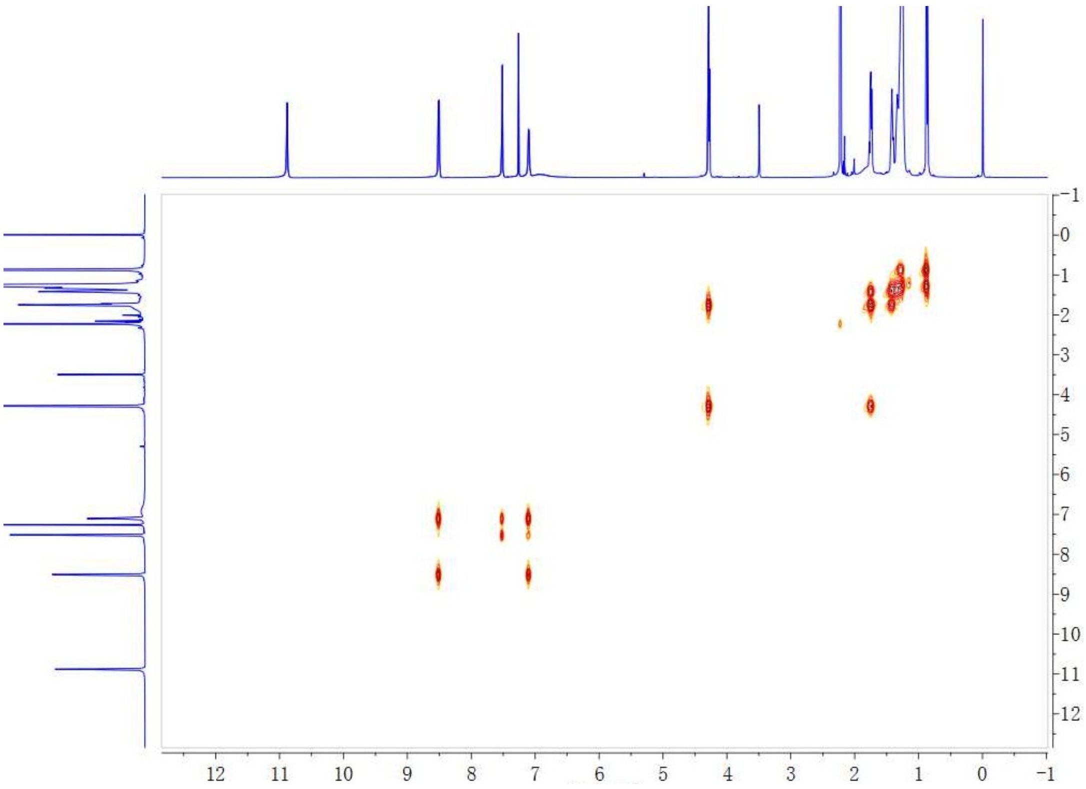

Figure S12. HMBC spectrum of compound **2** in $CDCl_3$

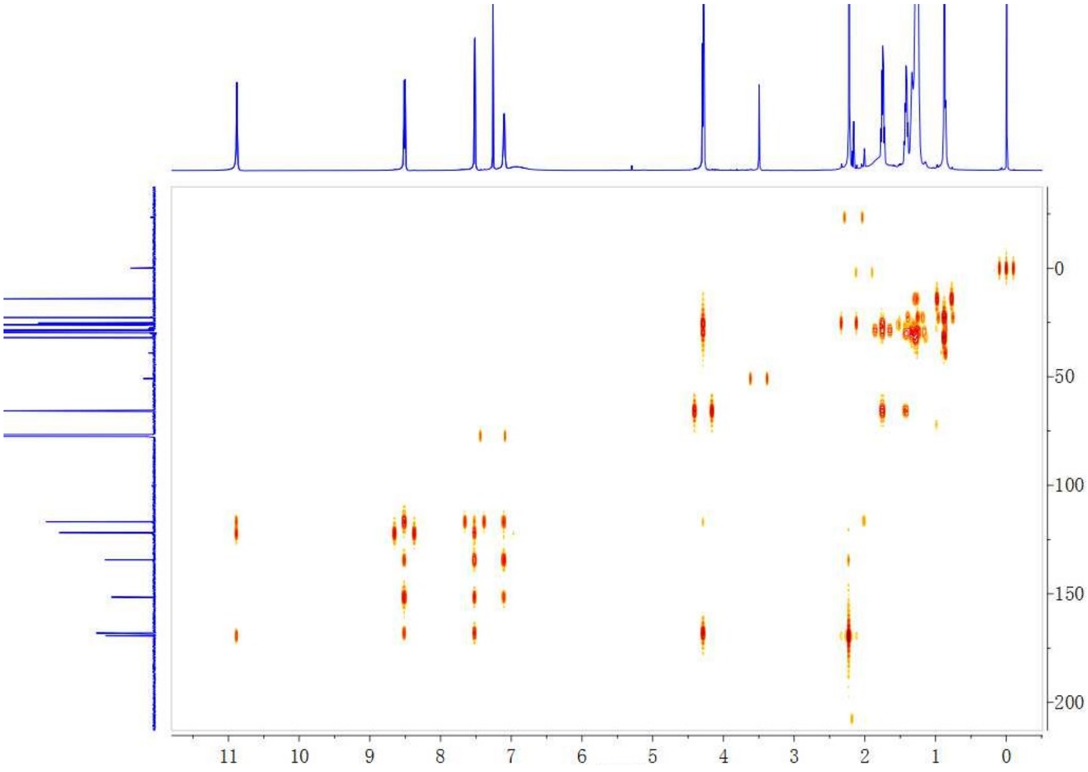

Figure S13. ^{1}H-NMR (600 MHz, $CDCl_3$) spectrum of compound **3**

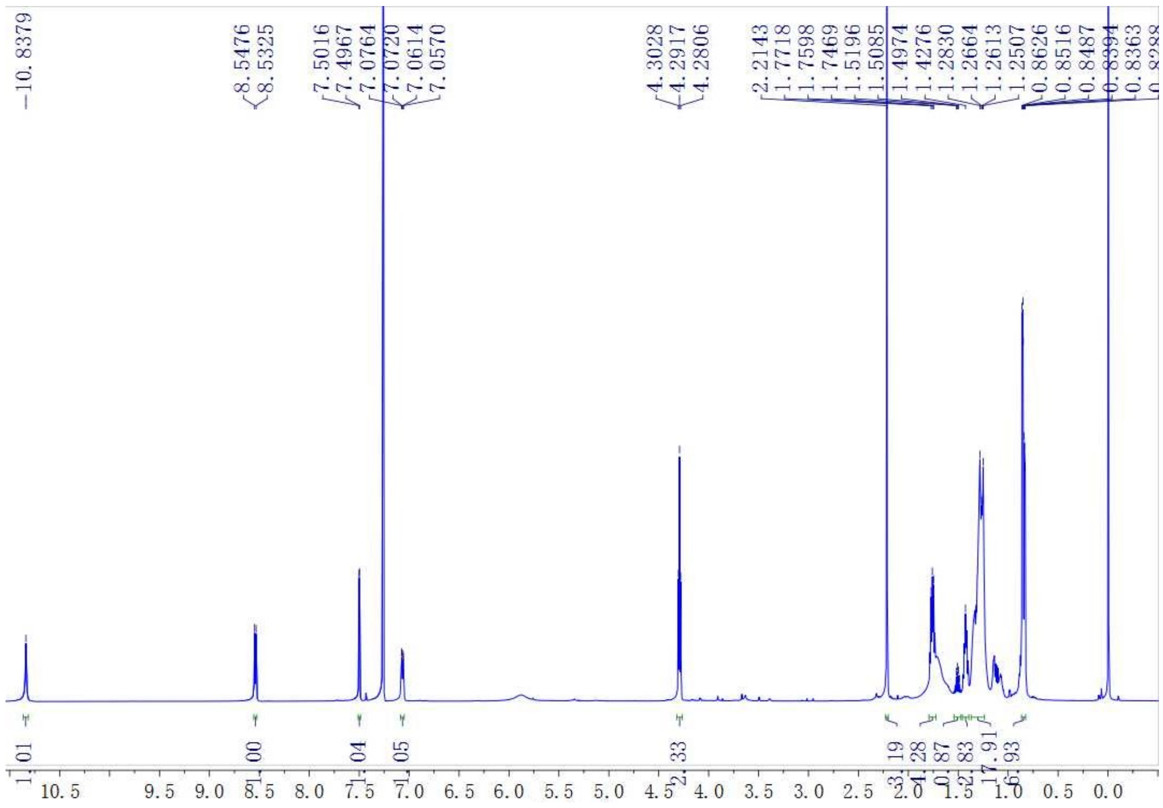

Figure S14. ^{13}C-NMR (150 MHz, $CDCl_3$) spectrum of compound **3**

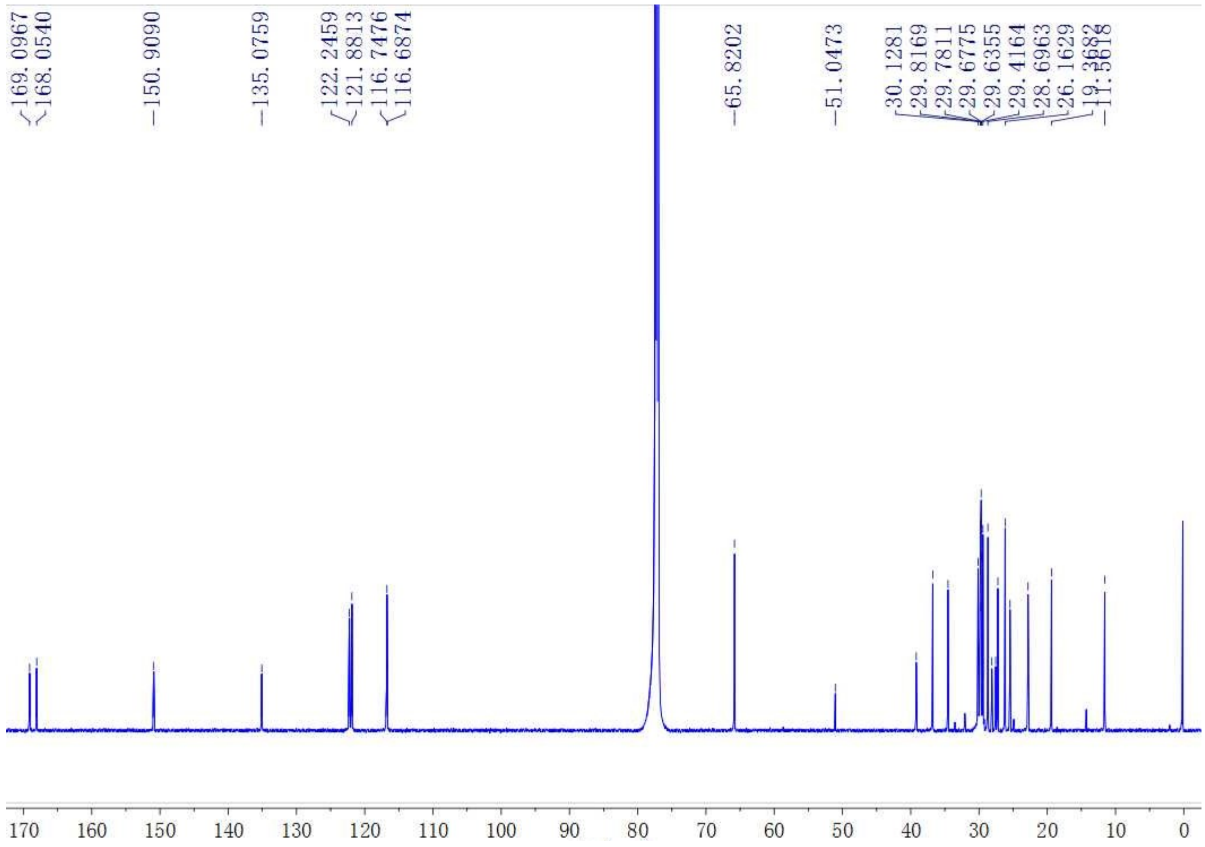

Figure S15. HSQC spectrum of compound **3** in $CDCl_3$

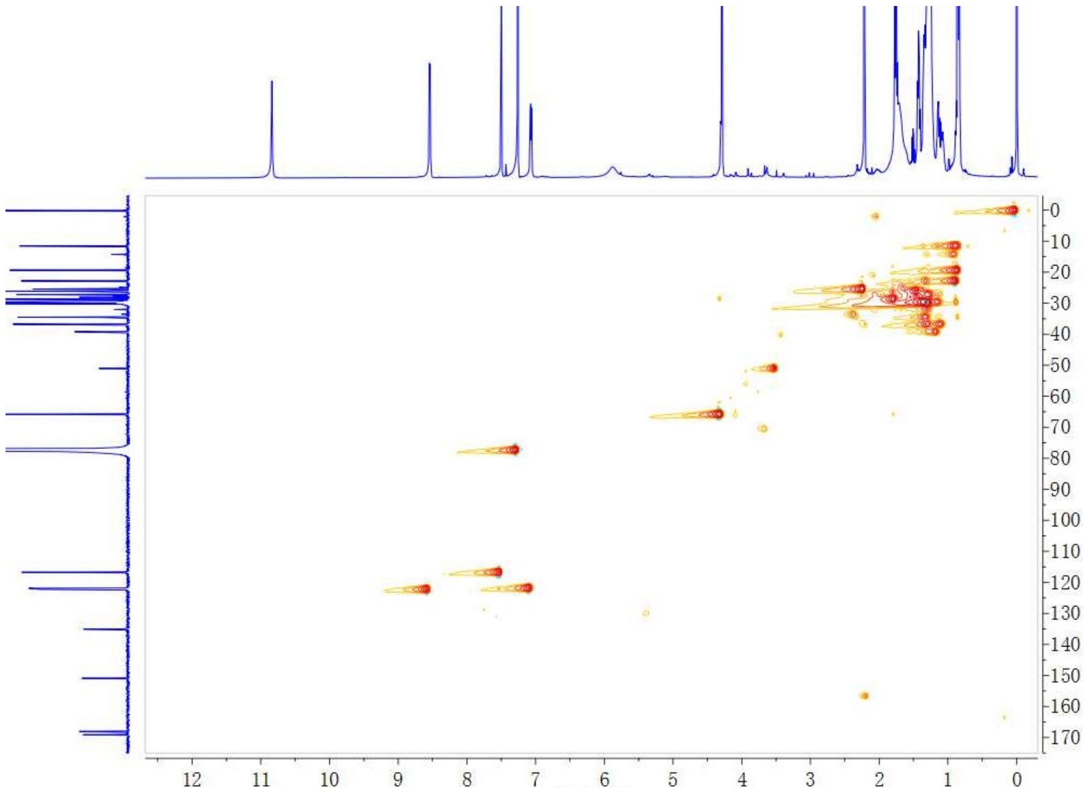

Figure S16. ^{1}H-NMR (600 MHz, $CDCl_3$) spectrum of compound **4**

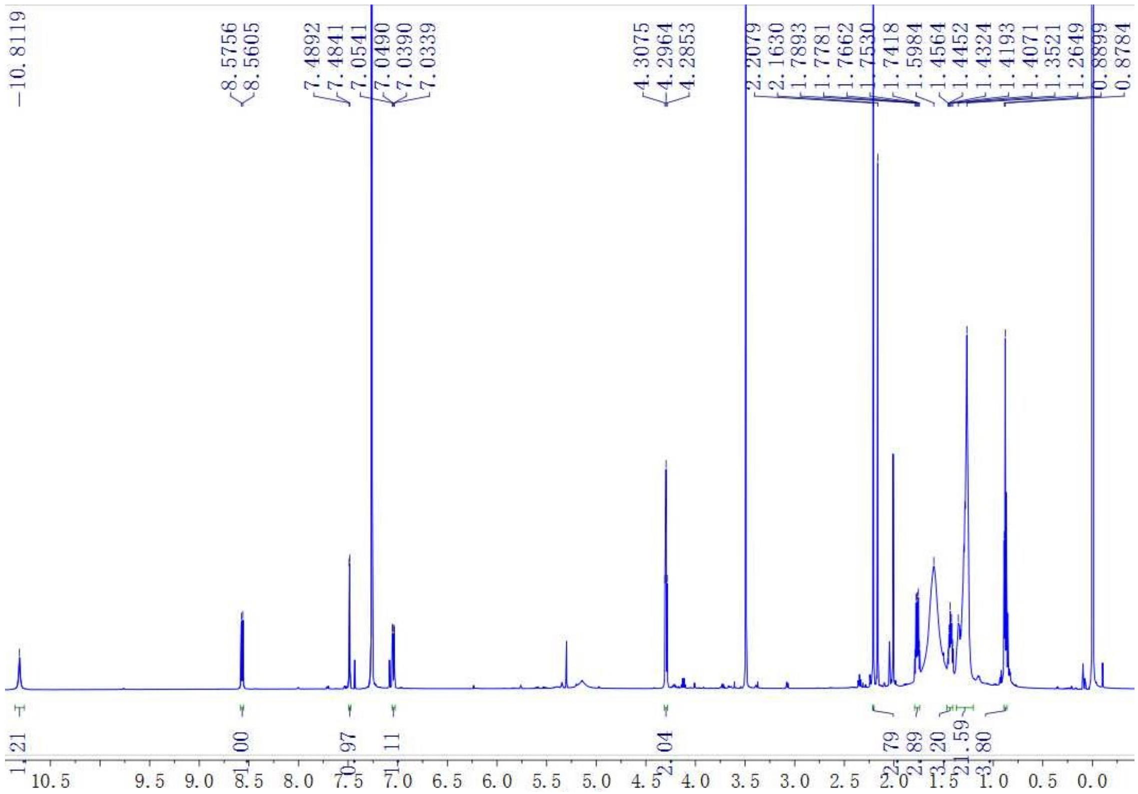

Figure S17. ^{13}C-NMR (150 MHz, $CDCl_3$) spectrum of compound **4**

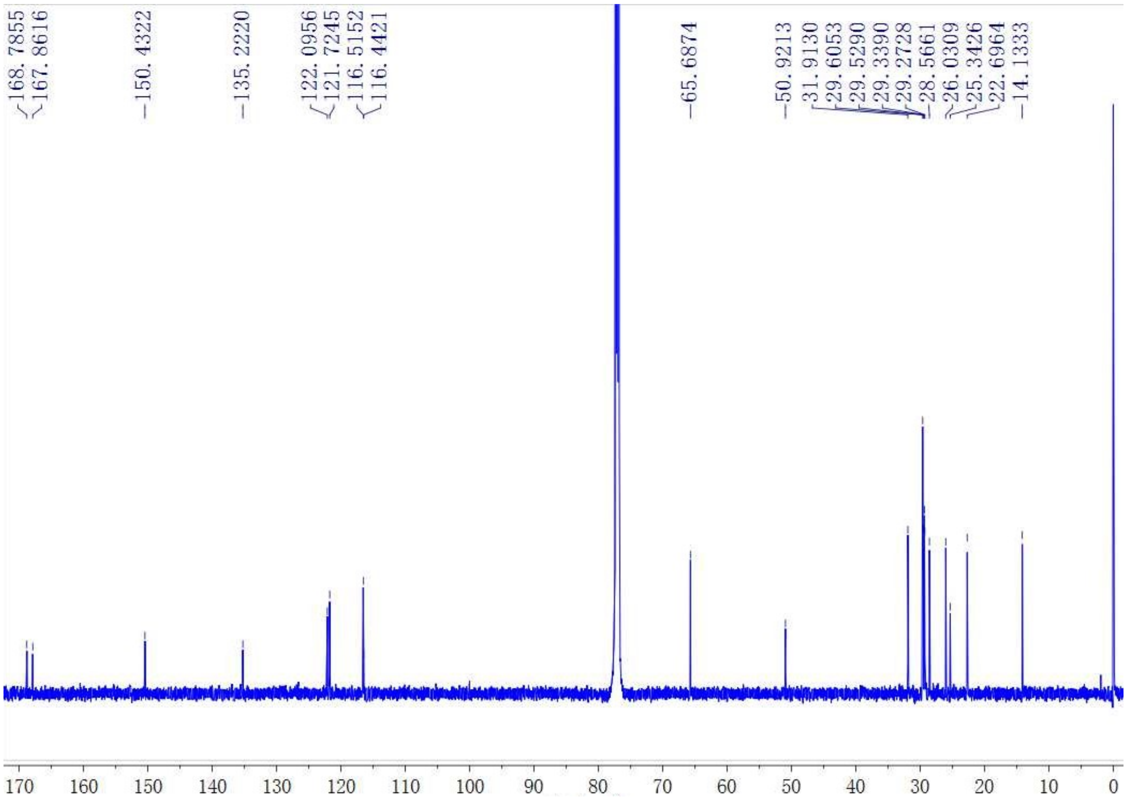

Figure S18. DEPT135 spectrum of compound **4** in $CDCl_3$

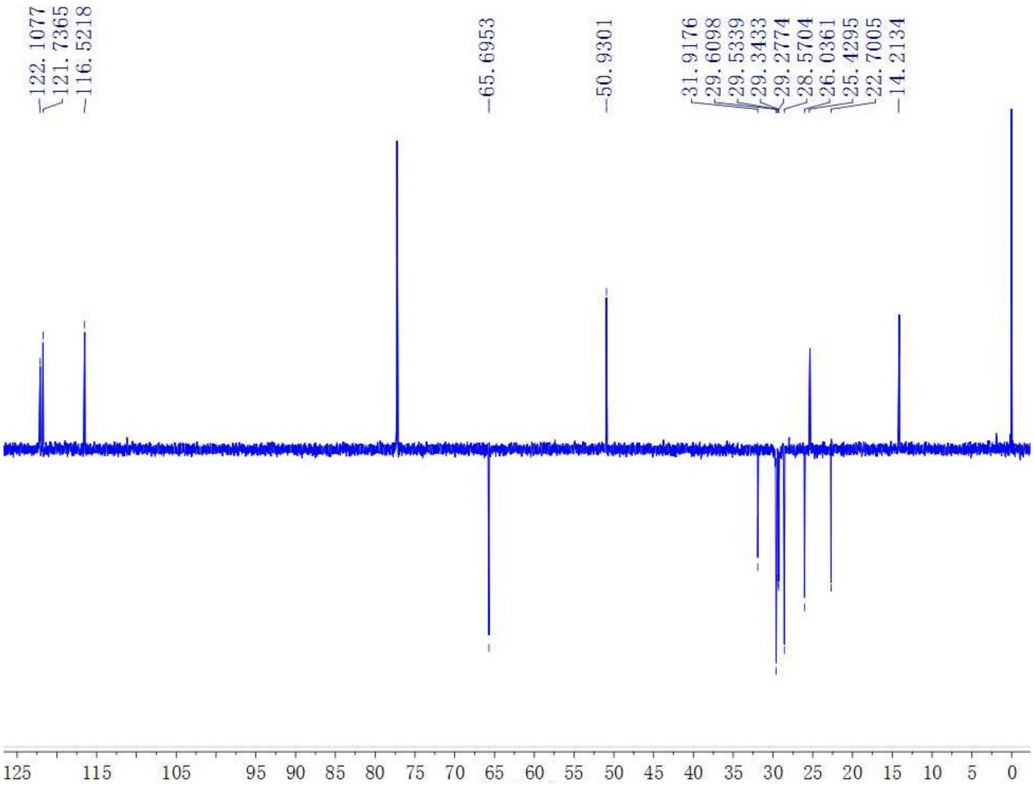

Figure S19. HSQC spectrum of compound **4** in $CDCl_3$

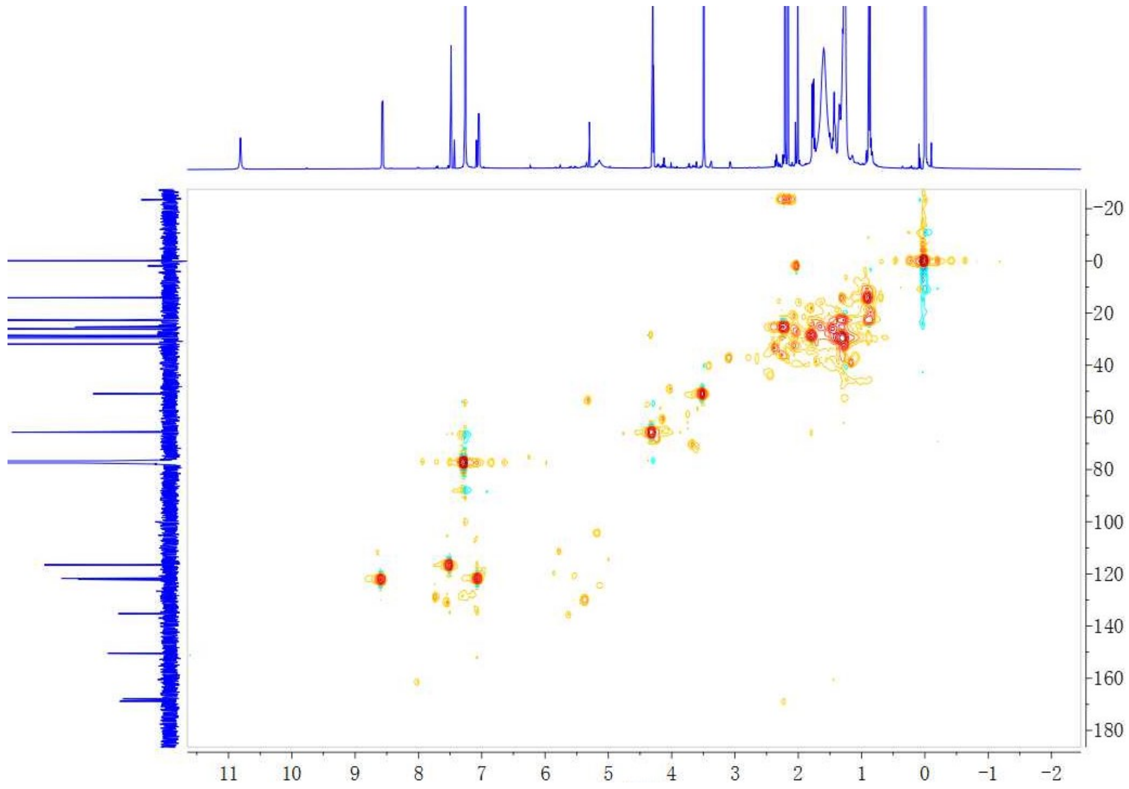

Figure S20. ^{1}H-^{1}H COSY spectrum of compound **4** in $CDCl_3$

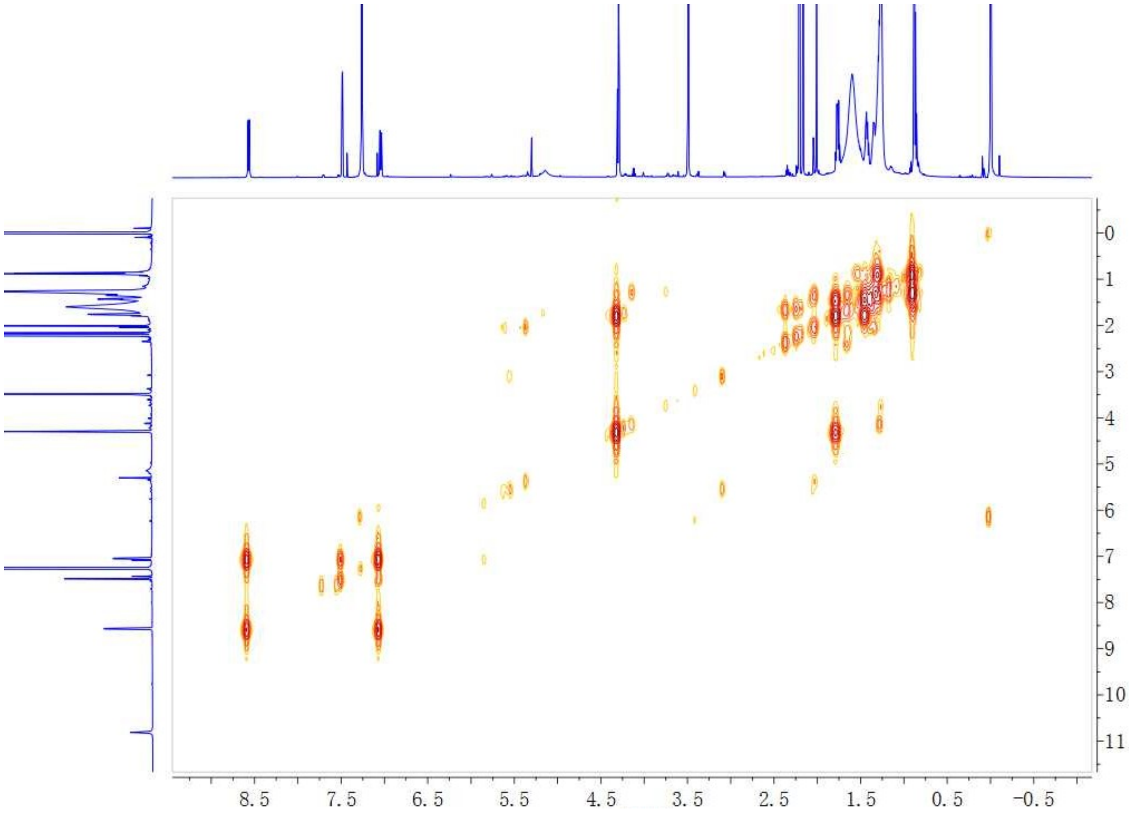

Figure S21. HMBC spectrum of compound **4** in $CDCl_3$

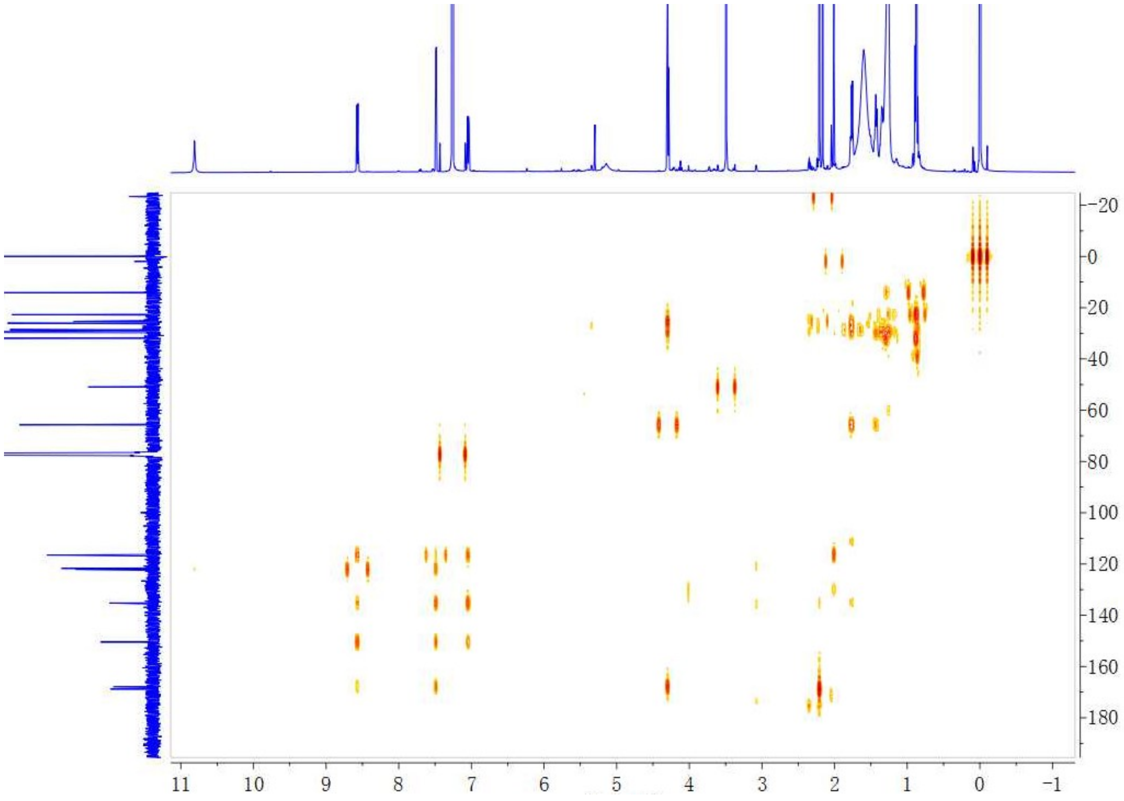

Supplement: Supplementary file 1 [file molecules-23-01032-s001.pdf]
